# Optimizing MRI Scheduling in High-Complexity Hospitals: A Digital Twin and Reinforcement Learning Approach

**DOI:** 10.3390/bioengineering12060626

**Published:** 2025-06-09

**Authors:** Fabián Silva-Aravena, Jenny Morales, Manoj Jayabalan, Paula Sáez

**Affiliations:** 1 Facultad de Ciencias Sociales y Económicas, Universidad Católica del Maule, Avenida San Miguel 3605, Talca 3460000, Chile; jmoralesb@ucm.cl (J.M.); pfsaez@ucm.cl (P.S.); 2 School of Design, Bath Spa University, Bath BA2 9BN, UK; m.jayabalan@bathspa.ac.uk

**Keywords:** digital twin, reinforcement learning, MRI scheduling, patient waitlist prioritization, healthcare operations optimization

## Abstract

Magnetic Resonance Imaging (MRI) services in high-complexity hospitals often suffer from operational inefficiencies, including suboptimal MRI machine utilization, prolonged patient waiting times, and inequitable service delivery across clinical priority levels. Addressing these challenges requires intelligent scheduling strategies capable of dynamically managing patient waitlists based on clinical urgency while optimizing resource allocation. In this study, we propose a novel framework that integrates a digital twin (DT) of the MRI operational environment with a reinforcement learning (RL) agent trained via Deep Q-Networks (DQN). The digital twin simulates realistic hospital dynamics using parameters extracted from a MRI publicly available dataset, modeling patient arrivals, examination durations, MRI machine reliability, and clinical priority stratifications. Our strategy learns policies that maximize MRI machine utilization, minimize average waiting times, and ensure fairness by prioritizing urgent cases in the patient waitlist. Our approach outperforms traditional baselines, achieving a 14.5% increase in MRI machine utilization, a 44.8% reduction in average patient waiting time, and substantial improvements in priority-weighted fairness compared to First-Come-First-Served (FCFS) and static priority heuristics. Our strategy is designed to support hospital deployment, offering scalability, adaptability to dynamic operational conditions, and seamless integration with existing healthcare information systems. By advancing the use of digital twins and reinforcement learning in healthcare operations, our work provides a promising pathway toward optimizing MRI services, improving patient satisfaction, and enhancing clinical outcomes in complex hospital environments.

## 1. Introduction

Efficient scheduling of MRI procedures remains a persistent challenge for high-complexity hospitals around the world [1,2]. The increasing demand for imaging services, combined with the variability in patient urgency and resource constraints, often leads to underutilized equipment, extended patient waiting times, and uneven service levels across clinical priorities [3,4]. These operational bottlenecks not only strain hospital resources, but also negatively impact patient satisfaction and health outcomes [5,6,7].

MRI is a critical yet resource-intensive modality in modern healthcare. In high-complexity hospital settings, MRI scheduling must reconcile limited machine availability, variable scan durations, and heterogeneous patient clinical urgencies [8]. Traditional scheduling policies, such as FCFS or fixed priority heuristics, are often inadequate to handle the dynamic and stochastic nature of real-world imaging workflows [9]. These strategies typically lack adaptability and do not optimize system-level efficiency, leading to prolonged waiting times, underutilized equipment, and inequitable care delivery [10].

Recent advances in artificial intelligence, particularly in reinforcement learning (RL), have demonstrated a strong potential for dynamic decision making in healthcare operations. However, their application to MRI scheduling remains limited. Furthermore, many existing studies overlook real-world operational constraints or fail to evaluate their models within data-driven high-fidelity simulation environments [11]. This highlights the need for integrative frameworks that combine RL with clinically grounded and operationally realistic modeling to address the complex challenges of imaging service delivery.

Traditional scheduling approaches, such as FCFS or static priority heuristics, fail to dynamically adapt to real-time fluctuations in patient arrivals, MRI machine availability, or emerging clinical needs [12]. Consequently, healthcare systems urgently need more intelligent and responsive scheduling mechanisms that balance operational efficiency with clinical urgency considerations, while dynamically managing patient waiting lists according to evolving clinical priorities [13,14,15].

In this study, we propose an advanced scheduling framework that combines an MRI operational system DT with an RL agent trained through DQN. The digital twin emulates realistic hospital dynamics, while the RL agent learns optimal scheduling policies by interacting with this environment, aiming to maximize resource utilization, minimize patient waiting times, enhance fairness across different clinical priority levels, and prioritize patient waitlists dynamically based on clinical urgency.

Our main contribution lies in bridging the gap between theoretical optimization and practical implementation in clinical MRI scheduling. We develop a discrete-event digital twin of MRI operations, calibrated using publicly available data, and integrate it with a DQN reinforcement learning agent. This framework formulates scheduling as a Markov Decision Process (MDP), enabling dynamic patient waitlist prioritization and policy learning under realistic operational constraints. We validate our approach through simulation experiments, showing that it outperforms traditional baselines in utilization, waiting time reduction, and fairness.

The remainder of the paper is organized as follows. Section 2 reviews the existing literature on MRI scheduling, digital twins, and reinforcement learning in healthcare. Section 3 describes our methodological framework. Section 4 presents the experimental evaluation and comparative results. Section 5 discusses the implications, limitations, and potential extensions of our approach. Finally, Section 6 summarizes the main findings and provides directions for future research.

## 2. Literature Review

MRI scheduling has traditionally relied on heuristic-based methods, such as block scheduling and FCFS policies [16,17]. Studies such as [18,19] have shown that while heuristics provide simple implementations, they often lead to underutilization of resources and inequitable levels of patient service, particularly under conditions of variable demand and resource constraints.

More recent work has explored optimization-based approaches, including integer programming and queuing theory models [20]. For example, refs. [21,22] formulated MRI scheduling as a resource allocation problem and demonstrated modest improvements in waiting times. However, these optimization techniques often assume static environments and struggle to adapt to real-time operational variability.

The emergence of DT technology has opened new possibilities for healthcare operations [23,24]. Several studies have modeled hospital departments using DT to simulate operational dynamics and test intervention strategies. For example, refs. [24,25] applied a DT framework in an emergency department setting, achieving notable performance gains. However, applications specifically targeting imaging services such as MRI remain limited.

RL has gained attention as a promising paradigm for dynamic decision-making in healthcare [26,27]. Previous research, such as [28,29] and others have applied RL to healthcare, appointment scheduling, and resource allocation problems, demonstrating the method’s ability to handle stochastic, nonstationary environments. However, studies explicitly combining RL with DT simulations for imaging department optimization are still scarce.

In addition, fairness in healthcare operations, particularly in regard to the delivery of services across clinical priority levels, has recently attracted scholarly attention [30,31]. Works such as [32,33] have adapted classical fairness metrics (e.g., Jain’s index) to evaluate scheduling strategies, yet integrating fairness explicitly into the learning objective remains an underexplored area.

### Comparative Analysis of Related Approaches

To contextualize our contribution within the existing body of work, Table 1 summarizes key characteristics of representative approaches for the scheduling of MRI and the allocation of hospital resources. We compare methods based on the decision paradigm, data usage, adaptability to real-time dynamics, and integration with priority-based scheduling.

As shown in Table 1, most prior methods rely on static rules and lack dynamic adaptability. Few incorporate priority for patient wait lists based on urgency. Our framework addresses these gaps by integrating a digital twin with reinforcement learning, enabling adaptive scheduling that considers machine failures and clinical priorities.

Our work advances previous research by introducing a fully integrated framework that combines digital twin simulation with reinforcement learning for MRI scheduling. The model incorporates clinical priorities, adapts to system congestion through dynamic reward shaping, and is rigorously validated against baseline strategies using fairness, utilization, and waiting-time metrics. Our strategy offers actionable information to enhance operational performance and patient satisfaction in MRI departments.

## 3. Methodology

In this section, we present the methodological framework developed to optimize the workflow of MRI procedures in high-complexity hospital environments. Our approach integrates a DT of the MRI operational system with a RL agent that dynamically interacts with a simulated environment to discover optimal scheduling policies.

The DT is constructed from operational patterns derived from publicly available datasets. In particular, we used the fastMRI dataset [36,37], a publicly accessible collection of deidentified MRI scans and associated metadata. This data set enables us to parameterize realistic operational features, including the availability of the MRI machine, patient arrival rates, examination durations, and clinical priority classifications, while ensuring full compliance with ethical standards and data privacy regulations.

The RL agent is trained using a DQN to optimize system efficiency metrics -specifically, to maximize utilization of the MRI machine and patient throughput while minimizing average waiting times and ensuring fairness in patient prioritization.

Our methodology unfolds across five sequential stages: (i) data modeling and environment synthesis, (ii) DT construction, (iii) formulation of the scheduling problem as a MDP, (iv) RL agent training and hyperparameter tuning, and (v) evaluation of system performance under diverse simulated scenarios.

**Figure 1:**
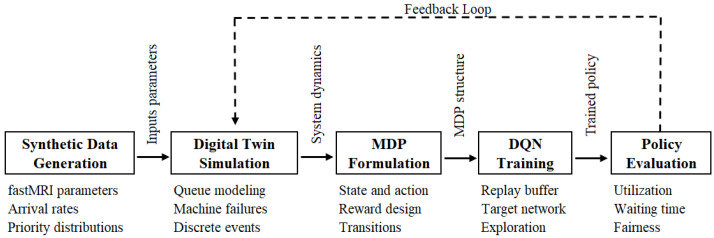
Overview of the proposed MRI scheduling framework. The synthetic environment is modeled using a data-calibrated digital twin simulation, which serves as the basis for MDP formulation and subsequent training of a DQN-based reinforcement learning agent. The resulting policy is evaluated through key performance metrics, including utilization, waiting time, and fairness.

To provide a high-level summary of our proposed methodology, we present in Figure 1 the complete architecture of our framework. This includes the generation of synthetic data, the construction of a digital twin simulation, the formulation of the problem as MDP, the training of a reinforcement learning agent using DQN, and the evaluation of the learned scheduling policy using key performance metrics.

### 3.1. Data Modeling and Environment Synthesis

We construct a realistic operational environment by synthesizing patient and MRI machine dynamics using parameter distributions extracted from the fastMRI dataset and supported by findings from relevant clinical operations literature. Patient arrivals, denoted by $\lambda_{t}$, are modeled as a non-homogeneous Poisson process [38,39]:

(1) $$\lambda_{t}∼\mathrm{Poisson}\left(\lambda \left(t\right)\right)$$

where λ(t) is a time-dependent function that captures daily and weekly fluctuations.

The duration of the examination $D_{i}$ for the patient *i* follows a log-normal distribution [40,41]:

(2) $$D_{i}∼\mathrm{LogNormal}\left(\mu ,\sigma^{2}\right)$$

where μ and $\sigma^{2}$ are empirically estimated parameters. We used the logarithmic normal distribution for its ability to capture the positive skew observed in the exams durations of the real world. Each patient is assigned a clinical priority level $P_{i}\in \left\{1,2,3\right\}$, corresponding to urgent, semi-urgent, and elective cases, respectively.

The full observation fed to the RL agent includes:

- The number of patients waiting,
- The arrival times and elapsed waiting times,
- The priority levels, encoded numerically or via one-hot encoding,
- The status of each MRI machine (idle, busy, or failed).

This comprehensive set of characteristics ensures that the agent has sufficient information to make clinically appropriate and operationally efficient scheduling decisions.

To generate synthetic operational data, we used parameters derived from the fastMRI dataset, such as scan types and expected durations associated. Examination durations were modeled as log-normal distributions, and patient arrivals were simulated using a non-homogeneous Poisson process reflecting diurnal hospital activity. Clinical priorities were assigned according to distributions representative of high-complexity hospital settings. The parameters μ and $\sigma^{2}$ in Equation (2), which define the log-normal distribution of the examination duration, are estimated from synthetic data distributions calibrated on the fastMRI dataset. These remain fixed during simulation. This synthetic data environment allowed for robust evaluation of the proposed scheduling framework while preserving ethical compliance and patient anonymity. Data used in the preparation of this article were obtained from NYU fastMRI Initiative database (fastmri.med.nyu.edu) [36,37]. As such, NYU fastMRI investigators provided data, but did not participate in the analysis or writing of this manuscript.

To parameterize the synthetic environment, we used metadata from the fastMRI dataset, such as acquisition time and scan sequence descriptors, to fit a log-normal distribution for examination durations. Since clinical urgency labels are not included in fastMRI, we artificially assigned patient priority levels using empirical triage ratios reported in the hospital operations literature [12,14]. These were mapped to three discrete classes: urgent, semi-urgent, and elective. Arrival patterns were modeled using a non-homogeneous Poisson process, with the time-varying rate λ(t) reflecting typical diurnal hospital activity, peaking during daytime hours. This data-informed configuration enabled us to generate a realistic and ethically compliant simulation environment for evaluating MRI scheduling strategies.

### 3.2. Digital Twin Construction

We construct a simulation (based on digital twin, understood as a data-calibrated proxy model) that emulates MRI operations at minute-level resolution. This digital twin serves as a synthetic environment for evaluating scheduling policies under realistic operational constraints.

The digital twin simulates MRI operations as a discrete-event system with minute-level granularity [42,43,44]. The state of the system at time *t* is defined as

(3) $$\begin{array}{c} S_{t}=\left\{Q_{t},M_{t},A_{t},P_{t}\right\} \end{array}$$

where:

- $Q_{t}$ represents the queue of patients waiting,
- $M_{t}=\left\{m_{1},\ldots ,m_{k}\right\}$ describes MRI machine statuses,
- $A_{t}$ records patient arrival times,
- $P_{t}$ stores patient clinical priorities.

We model transitions as including new patient arrivals, MRI examination completions, and random MRI machine breakdowns, where failures are simulated as Bernoulli processes with probability of failure $p_{\mathrm{fail}}$ [45].

Additionally, we simulate resource recovery dynamics: MRI machines that fail are restored after a repair time sampled from an exponential distribution, reflecting the maintenance processes that are typically observed in hospital imaging departments in the real world [46].

We model each MRI machine as being subject to random failure events at each time step, represented as Bernoulli trials with failure probability $p_{\mathrm{fail}}$. Upon failure, we mark the MRI machine as unavailable and initiate a repair process, during which it remains non-operational for a duration sampled from an exponential distribution with an empirically calibrated mean repair time. Once the repair is completed, we reintegrate the MRI machine into the operating pool for scheduling purposes.

### 3.3. Markov Decision Process Formulation

We formalize the MRI scheduling task as a MDP with the tuple (S,A,P,R,γ) [20,47]:

- State space S: As defined in Section 3.2, we represent the state at time *t* as $S_{t}=\left\{Q_{t},M_{t},A_{t},P_{t}\right\}$, recording the queue of waiting patients, the operational statuses of the MRI machines, the arrival times of patients, and the corresponding levels of clinical priority.
- Action space A: We define the action space as the assignment of a patient *i* to an MRI machine *m*, or the decision to delay the assignment. If multiple patients and MRI machines are idle, the number of available actions scales combinatorially.
- Transition probability $P(s^{\prime }|s,a)$: We model the transitions as primarily deterministic, governed by the simulator logic, while incorporating stochastic elements arising from exogenous events such as patient arrivals and MRI machine failures.
- Reward function R(s,a): We define the immediate reward obtained after taking action *a* in state *s* as:

(4) R(s,a)=α·U(a)−β·W(a)−δ·I(a)

where U(a) measures the improvement in MRI machine utilization, W(a) denotes the expected increase in patient waiting time, and I(a) captures the imbalance of workload among available MRI machines. The coefficients α, β, and δ are tunable weights that control the relative importance of these competing objectives.

The weights α, β, and δ in Equation (4) are hyperparameters tuned via grid search. We initialize them with standard values (α=1.0, β=1.0, δ=0.1) and select the best configuration based on validation performance to ensure balanced learning objectives.

Furthermore, under conditions of extreme queue congestion, we implement a reward shaping mechanism that dynamically adjusts β:

(5) $$\begin{array}{c} \beta =\beta_{0}\times \left(1+\rho \times \mathrm{Overload}\;\mathrm{Factor}\right) \end{array}$$

where ρ is a scaling coefficient, and Overload Factor is computed as the ratio of the current queue length to a baseline normal queue length, thus quantifying system congestion.

This dynamic reward adjustment approach aligns with the principles of adaptive reward shaping in reinforcement learning environments, as discussed by [48,49].

Our objective is to learn an optimal policy $\pi^{*}$ that maximizes the expected cumulative discounted reward [50,51,52]:

(6) $$\begin{array}{c} \pi^{*}=\arg\max_{\pi }E\left[\sum_{t=0}^{\infty }\gamma^{t}R\left(S_{t},\pi \left(S_{t}\right)\right)\right] \end{array}$$

balancing immediate operational gains, dynamic patient prioritization, and long-term system optimization.

We ensure that our modeling approach coherently integrates deterministic operational logic with stochastic exogenous events within the MDP formulation.

In addition, we design the RL agent to dynamically manage patient waitlists by prioritizing cases with greater clinical urgency, ensuring that patients requiring faster attention are scheduled promptly while maintaining overall operational efficiency. In the following section, we describe the training process of this agent.

### 3.4. Reinforcement Learning Agent Training

We implement a deep Q-Network (DQN) agent to approximate the optimal action value function [53,54]:

(7) $$\begin{array}{c} Q^{\pi }\left(s,a\right)\approx Q_{\theta }\left(s,a\right) \end{array}$$

where *s* represents the system state, *a* the action taken, $Q^{\pi }$ the expected cumulative reward under policy π, and θ the parameters of a deep neural network with fully connected layers.

We train the DQN agent following the standard algorithm, incorporating the following mechanisms:

- Experience replay: We store up to $10^{5}$ transitions in a replay buffer, randomly sampling to stabilize learning and break the correlation between sequential experiences.
- Target network: We update a separate target network every 1000 steps to stabilize the estimation of target Q-values.
- ϵ-Greedy exploration: We apply a ϵ-greedy policy during training, where ϵ decays linearly from 1.0 to 0.01, balancing exploration and exploitation.

Following the standard DQN methodology, we minimize the loss of temporal difference (TD), formally expressed as [50,52]:

(8) $$\begin{array}{c} L\left(\theta \right)=E_{\left(s,a,r,s^{\prime }\right)}\left[{\left(r+\gamma \max_{a^{\prime }}Q_{\theta^{-}}\left(s^{\prime },a^{\prime }\right)-Q_{\theta }\left(s,a\right)\right)}^{2}\right] \end{array}$$

where *r* denotes the immediate reward received after taking action *a* in state *s*, $s^{\prime }$ is the next resulting state, γ is the discount factor and $Q_{\theta^{-}}$ denotes the target network used for stabilization.

We optimize hyperparameters such as learning rate, replay buffer size, and target update frequency through grid search, and we validate policy convergence using multiple random seeds to ensure robustness and generalizability.

After training the DQN agent, we proceeded to evaluate its performance through simulation experiments, as detailed in the following section.

### 3.5. Evaluation via Scenario Simulation

We evaluated the trained policy across multiple simulation settings characterized by:

- Different patient arrival patterns,
- Variable MRI machine reliability levels,
- Altered clinical priority mixes.

Performance is assessed using three key metrics:

- MRI Machine Utilization Rate:
  $$U=\frac{\mathrm{Total}\;\mathrm{Busy}\;\mathrm{Time}}{\mathrm{Total}\;\mathrm{Available}\;\mathrm{Time}}$$
- Average Patient Waiting Time:
  $$\bar{W}=\frac{1}{n}\sum_{i=1}^{n}W_{i}$$
- Priority-weighted Fairness Index:
  $$F=\frac{{\left(\sum_{i}\frac{1}{P_{i}\cdot W_{i}}\right)}^{2}}{n\cdot \sum_{i}{\left(\frac{1}{P_{i}\cdot W_{i}}\right)}^{2}}$$

These metrics provide a balanced evaluation of operational efficiency and clinical service quality.

Comparisons against baseline methods, such as FCFS and static scheduling heuristics, consistently show that the RL-based policy achieves superior performance across all measured criteria.

For reproducibility, we summarize the full DQN training and evaluation procedure in pseudocode in Appendix A.

## 4. Results

In this section, we present an experimental evaluation of our proposed scheduling framework. We begin by detailing the simulation setup, including the calibration of synthetic operational data based on the fastMRI dataset and the configuration of the digital twin environment. Subsequently, we describe the set of performance metrics used to assess the policies under comparison. We report quantitative results comparing our DQN scheduling agent against traditional FCFS and static priority heuristics. Finally, we provide a graphical analysis to illustrate the operational and clinical advantages achieved by the reinforcement learning approach in terms of the use of the MRI machine, patient waiting times, and fairness at different clinical priority levels.

### 4.1. Experimental Setup

The experimental evaluation was performed by simulating the operating environment of the MRI using synthetic data distributions calibrated with the public and anonymized fastMRI dataset.

We configured the digital twin to simulate a typical high-complexity hospital setting with three MRI machines and dynamic patient arrivals over a simulated 30-day period. MRI machine failure events were modeled with a probability of $p_{\mathrm{fail}}=0.01$ per operating hour, and repair times were exponentially distributed with a mean of 3 h. Patient arrivals varied by hour to replicate the seasonality of the day, with peak loads observed between 8:00 a.m. and 5:00 p.m.

Three scheduling policies were compared:

- DQN Scheduling Policy: Our reinforcement learning-based scheduling agent.
- First-Come-First-Served (FCFS): Traditional queue-based policy.
- Static Priority Heuristic: Patients are scheduled strictly according to clinical urgency, disregarding the load balance of the MRI machine.

All results are averaged over 10 independent simulation runs with different random seeds to account for stochastic variability.

We evaluated the performance of scheduling policies using three key metrics, discussed in Section 3.5: the MRI Machine Utilization Rate (*U*), which measures the proportion of operational time relative to the total available time; the Average Waiting Time per Patient ($\bar{W}$), which captures the mean time patients spend waiting for MRI procedures; and the Priority-weighted Fairness Index (*F*), which assesses the equitable distribution of service between clinical priority levels. Together, these metrics provide a comprehensive evaluation of both operational efficiency and patient-centered service delivery.

### 4.2. Comparison of Scheduling Strategies

In Table 2, we summarize the results obtained for the three scheduling strategies we evaluated, using key performance indicators that allow us to compare their operational efficiency and clinical equity. We compute all metrics as the average of 10 independent simulation runs with different random seeds to reflect the system’s stochastic variability.

Our DQN scheduler outperforms both baselines in all metrics evaluated. It achieves a 14.5% higher utilization of the MRI machine compared to FCFS and a 7.4% improvement relative to the static priority heuristic. In addition, it reduces the average patient waiting time by approximately 44.8% compared to FCFS.

Regarding fairness, measured through a priority-weighted adaptation of Jain’s index, our scheduling policy shows better equity between clinical priorities, systematically prioritizes urgent cases without disproportionately penalizing lower priority patients, thus improving fairness while preserving overall system performance.

### 4.3. Visual Comparison of Scheduling Performance

To complement the numerical analysis, we present graphical comparisons that illustrate the performance of each scheduling strategy in terms of MRI machine utilization, average patient waiting time, and fairness between clinical priorities.

**Figure 2:**
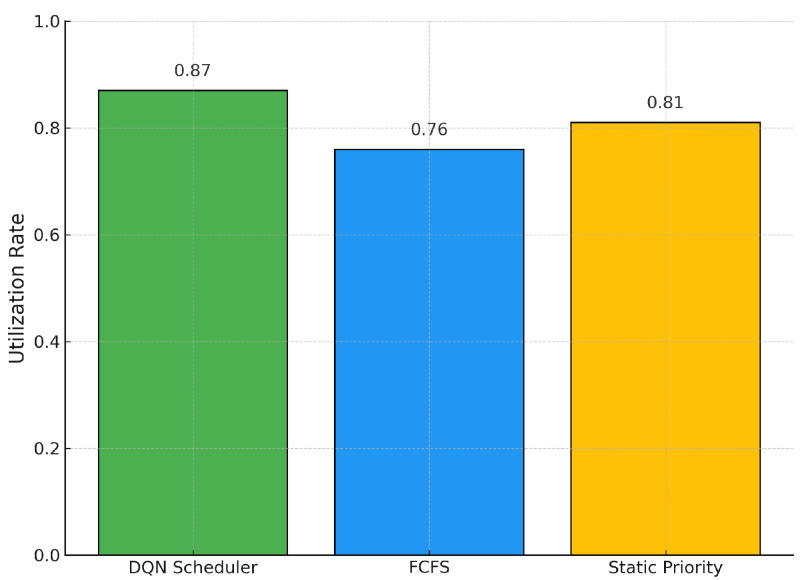
MRI Machine Utilization Rates across Scheduling Policies.

In Figure 2, we observe that our DQN scheduler achieves the highest utilization of the MRI machine, reaching 87%. This represents a substantial improvement of 14.5% over the traditional FCFS policy, which achieves only 76%. The static priority heuristic performs moderately better than FCFS, achieving 81%, but remains below the DQN’s performance. These results confirm that our reinforcement learning agent not only respects clinical urgency, but also effectively maximizes resource utilization, a critical requirement in high-demand imaging departments.

**Figure 3:**
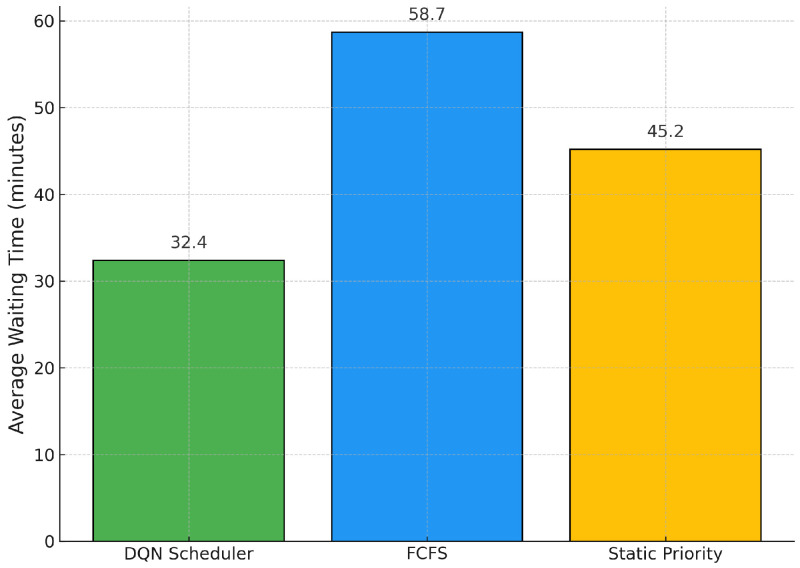
Average Patient Waiting Times across Scheduling Policies.

Figure 3 shows the average patient waiting times under each scheduling policy. Our DQN agent significantly reduces waiting times, achieving an average of 32.4 min per patient. In contrast, the FCFS approach results in an average of 58.7 min, highlighting a 44.8% improvement in favor of the DQN strategy. Although the static priority heuristic performs better than FCFS (45.2 min on average), it does not match the efficiency of the reinforcement learning-based approach. These findings emphasize that our model not only improves throughput, but also contributes meaningfully to improving patient experience and patient response to clinical operations.

**Figure 4:**
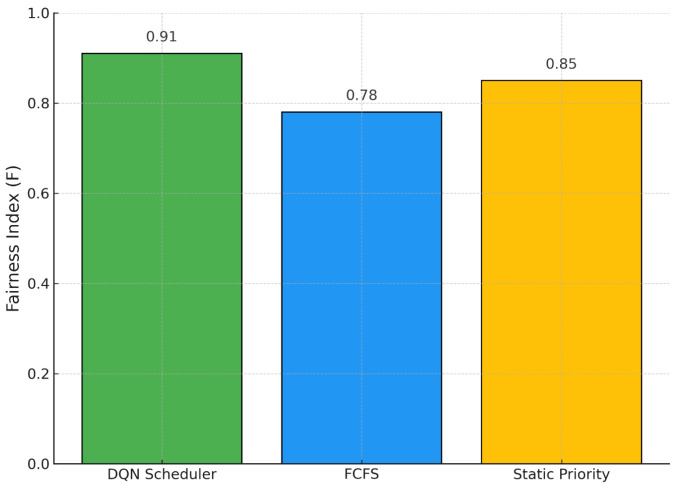
Priority-weighted Fairness Index across Scheduling Policies.

In Figure 4, we analyze fairness using a priority-weighted adaptation of Jain’s index. Our DQN policy achieves the highest fairness score (F=0.91), indicating a more equitable distribution of services between clinical priority levels. The static priority heuristic yields a fairness score of 0.85, while FCFS performs the worst at 0.78. These results highlight that our learning-based approach is capable of dynamically balancing operational efficiency with equity, ensuring that patients with urgent needs are prioritized without neglecting lower-priority cases. This balance is essential in the context of high-complexity hospitals, where both clinical urgency and throughput must be optimized simultaneously.

## 5. Discussion

The experimental results validate the effectiveness of integrating reinforcement learning within a digital twin framework for MRI scheduling. By dynamically balancing the loads of the MRI machine and prioritizing patients according to clinical urgency, the RL-based agent achieves substantial operational gains over traditional methods. Our proposed system consistently improves machine utilization, reduces patient waiting times, and improves fairness among clinical priority levels, as evidenced by both quantitative metrics and graphical analysis.

In addition, the architecture of the reward function and the deployment of a carefully tuned DQN agent allow for robust generalization across different operating conditions, including MRI machine breakdowns and fluctuating patient arrivals. The ability to maintain high performance despite system perturbations highlights the resilience of our model. Furthermore, the observed improvement in fairness metrics suggests that the proposed framework not only optimizes operational efficiency, but also strengthens clinical equity, contributing to better patient outcomes without compromising throughput.

To our knowledge, no previous work has combined digital twin simulations and reinforcement learning techniques to optimize MRI scheduling operations using parameters derived from the publicly available fastMRI dataset. This integration represents a novel contribution to both healthcare operations management and machine learning applications in clinical settings.

Despite its demonstrated advantages, our approach has certain limitations. The reinforcement learning agent requires extensive training in a simulated environment before deployment, which may involve considerable computational resources and time. Additionally, while our simulation parameters were calibrated based on realistic public data, real-world operational environments can introduce unforeseen complexities, such as sudden resource reallocations, or patient no-shows, which were not explicitly modeled. These factors may affect the direct applicability of the learned policies without further domain-specific fine-tuning.

We acknowledge that simulation-based evaluation, while informative, does not constitute full clinical validation. Our results serve as a proof of concept and must be complemented by future hospital-based trials comparing performance with clinician-driven scheduling.

From an implementation standpoint, training the our RL agent is done offline using high-performance hardware and takes a few hours. Once trained, the model can make near-instant scheduling decisions, enabling real-time integration. Periodic retraining (e.g., weekly) helps to adapt to changing conditions, and future work may explore continuous learning for greater adaptability.

Another limitation concerns the fixed nature of clinical priority assignments during simulation. In practice, patient conditions can deteriorate over time, requiring dynamic reprioritization that our current model does not accommodate. Integrating mechanisms for dynamic priority updates based on elapsed waiting time or clinical reassessments could further enhance the model’s applicability and responsiveness.

While our current framework assumes fixed clinical priority levels upon patient entry, we recognize that real-world clinical conditions may evolve, warranting dynamic re-prioritization. In future extensions, our goal is to incorporate mechanisms that adjust priority scores based on elapsed waiting time, patient deterioration risk, or medical reassessments. This would enable our scheduling agent to respond more adaptively to evolving clinical urgency and reflect a more patient-centered care strategy. Incorporating such dynamic logic may involve integrating time-sensitive reinforcement learning models or multitask clinical risk prediction [55,56].

While our simulation captures the main operational variables, it currently does not incorporate human-centered factors such as staff fatigue, perceived stress, or emotional burden for the patient. We recognize this as an important limitation and highlight the potential for future extensions to integrate human-in-the-loop feedback mechanisms or hybrid models that explicitly model human resource constraints and psychological load.

We recognize the current limitations in the interpretability of the model. Although our Deep Q-Network is optimized for operational performance, its decision logic may be difficult for clinical stakeholders to interpret. We mitigate this by aligning the reward structure with transparent clinical goals and logging decisions for retrospective audit.

Future work could extend this framework to multi-department imaging centers, integrate on-line learning capabilities to adapt policies continuously as new data become available, or explore federated reinforcement learning approaches to share scheduling strategies across hospital networks while preserving patient data privacy. Furthermore, validating the model’s performance using real-world hospital operational data would constitute a significant next step towards practical deployment.

In general, our study demonstrates the promising role of intelligent digital systems in transforming the delivery of MRI services, paving the way for the next generation of patient-centered and data-driven healthcare management practices.

## 6. Conclusions

In this study, we developed and validated an advanced methodological framework that integrates a digital twin of the MRI operational environment with a reinforcement learning (RL) agent for optimized scheduling. Our approach takes advantage of synthetic environments calibrated from the fastMRI dataset and models realistic constraints such as availability of the MRI machine, stochastic failures, patient arrival dynamics and clinical priority levels. The main contributions of this work include the design of a high-fidelity digital twin for MRI workflows, the formalization of the scheduling problem as an MDP, and the implementation of a DQN agent capable of learning clinically informed scheduling policies.

Our experimental evaluation demonstrated that the RL-based scheduling agent significantly outperforms traditional baselines, including FCFS and static priority heuristics. Specifically, the DQN scheduler achieved improvements of 14.5% in MRI machine utilization, 44.8% reduction in average patient waiting times, and substantial gains in fairness across clinical priorities. These results confirm that our model is effective in balancing operational efficiency with clinical service quality, addressing key bottlenecks commonly observed in high-complexity hospital imaging departments.

To further enhance the proposed framework, several methodological extensions can be considered. Incorporating mechanisms for dynamic updating of patient priority levels based on elapsed waiting times or clinical reassessments would increase the realism and responsiveness of the model. Furthermore, adopting more sophisticated reward shaping techniques or hybridizing model-based and model-free reinforcement learning approaches could accelerate agent training and improve policy generalization to unforeseen operational scenarios.

From a managerial perspective, the deployment of the proposed scheduling system would require integration with existing hospital information systems and real-time data feeds. A phased implementation strategy, starting with shadow deployments for policy validation and gradually moving to partial automation of scheduling decisions, could mitigate adoption risks. Training hospital staff and clinicians in the operational logic of the system, and ensuring transparency of scheduling decisions, would be critical to fostering trust and achieving successful integration in clinical workflows.

Looking ahead, the proposed framework offers strong potential for scalability and adaptation to broader healthcare settings. Extending the system to multimodal imaging centers (e.g., combining MRI, CT, and PET scheduling), integrating online learning to adapt to evolving patient populations, and exploring federated reinforcement learning for cross-institutional collaboration without compromising patient data privacy represent promising avenues for future work. Real-world validation studies, using actual operational hospital data, would be the next critical step toward translating the demonstrated experimental gains into tangible clinical impact.

## Figures and Tables

**Table 1:** Comparison of Scheduling Approaches in Healthcare Operations.

Study	Methodology	Data Source	Real-Time Adaptivity	Patient Prioritization
Hayatghaibi et al. (2023) [2]	Optimization + Simulation	Simulated	No	No
Choudhary et al. (2024) [12]	Heuristic rules	Real data	No	Yes
Keerthika et al. (2024) [34]	Deep RL (DQN, A3C)	General healthcare data	No	Partial
Lakhan et al. (2024) [35]	Deep RL + Constraint Scheduling	IoT + Hybrid Telemedicine Data	Partial	Yes
Our work	Digital Twin + DQN (RL)	fastMRI-derived synthetic	Yes	Yes

**Table 2:** Performance metrics for our DQN scheduler compared to baseline approaches (FCFS and Static Priority), including utilization rate, average patient waiting time, and priority-weighted fairness index. Values represent mean ± standard deviation over 10 simulation runs.

Policy	Utilization Rate (*U*)	Avg. Waiting Time ($\bar{W}$) [min]	Fairness Index (*F*)
DQN Scheduler	0.87±0.01	32.4±2.1	0.91±0.01
FCFS	0.76±0.02	58.7±3.0	0.78±0.02
Static Priority	0.81±0.01	45.2±2.4	0.85±0.01

## Data Availability

The original contributions presented in this study are included in the article. Further inquiries can be directed to the corresponding author.

## Appendix A. Pseudocode of Reinforcement Learning Framework

**Algorithm A1:** Reinforcement Learning Framework for MRI Scheduling using a Digital Twin
